# Prior Abdominal Surgery Jeopardizes Quality of Resection in Colorectal Cancer

**DOI:** 10.1007/s00268-015-3390-0

**Published:** 2016-01-13

**Authors:** Martijn W. J. Stommel, Johannes H. W. de Wilt, Richard P. G. ten Broek, Chema Strik, Maroeska M. Rovers, Harry van Goor

**Affiliations:** Department of Surgery, Radboud university medical center, P.O. Box 9101, 6500 HB Nijmegen, The Netherlands; Department of Operating Rooms and Health Evidence, Radboud university medical center, P.O. Box 9101, 6500 HB Nijmegen, The Netherlands

## Abstract

**Background:**

Prior abdominal surgery increases complexity of abdominal operations. Effort to prevent injury during adhesiolysis might result in less extensive bowel resection in colorectal cancer surgery. The aim of this study was to evaluate the effect of prior abdominal surgery on the outcome of colorectal cancer surgery.

**Methods:**

A nationwide prospective database of patients with primary colorectal cancer resection in The Netherlands between 2010 and 2012 was reviewed for histopathology, morbidity and mortality in patients with compared to patients without prior abdominal surgery.

**Results:**

9042 patients with and 17,679 without prior abdominal surgery were analyzed. After prior abdominal surgery 20.7 % had less than 10 lymph nodes in the histopathological specimen compared to 17.8 % without prior abdominal surgery (adjusted OR 1.17, 95 % CI 1.09–1.26). Adjusted ORs for less than 10 and 12 lymph nodes were significant in colon cancer resection and not in rectal cancer resection. Subgroups of patients who had previous hepatobiliary surgery or other abdominal surgery had a higher incidence of inadequate number of harvested lymph nodes. Prior colorectal surgery increased the percentage of positive circumferential rectal resection margin by 64 % (12.5 and 7.6 %; adjusted OR 1.70, 95 % CI 1.21–2.39). For colon cancer morbidity was significantly higher in patients with prior surgery (33.2 and 29.7 %; adjusted OR 1.18, 95 % CI 1.10–1.26), 30-day mortality was comparable (4.7 % prior surgery and 3.8 % without prior surgery; adjusted OR 1.01, 95 % CI 0.88–1.17).

**Conclusions:**

Prior abdominal surgery compromises the quality of resection and increases postoperative morbidity in patients with primary colorectal cancer.

## Introduction

Prior abdominal surgery increases the complexity and morbidity of abdominal operations, mainly due to the frequent presence of postoperative intra-abdominal adhesions [1]. The incidence of postoperative intra-abdominal adhesions after previous surgery ranges between 67 and 95 % [2–5]. Adhesions may necessitate adhesiolysis, which is time consuming and results in full-thickness or seromuscular bowel injury in one-third of the patients [6]. Adhesiolysis and associated bowel injury increase morbidity and mortality [6, 7]. The cautious approach to the bowel during adhesiolysis to avoid injury, might compromise access to the operative field and extent of bowel resection, with possibly smaller or even incomplete resection margins. When access to the pelvic area for performing rectal resection is difficult due to a previous operation, care is taken not to injure ureters or main vessels and nerves, possibly limiting mesorectal excision. A small resection margin, has been identified as a risk factor for poor lymph node harvest [8]. Minimal information is available regarding the effect of previous surgery on quality of resection margin or number of lymph nodes. Two retrospective studies showed no difference in number of harvested lymph nodes and resection margin [9, 10]. Both studies comprised low numbers of patients (*n* = 86 and *n* = 267), and both reported overall mean number of harvested lymph nodes less than 10. This low number possibly reflects inadequate surgical resection or inadequate quality of histopathological examination of the specimen.

Since 2009 the Dutch Surgical Colorectal Audit (DSCA) database is in use to assess and benchmark outcomes of colorectal cancer treatments between hospitals. Prior surgery is one of the many variables recorded in this database. This gave us the opportunity to evaluate the effect of prior abdominal surgery on the outcome of colorectal cancer surgery in more than 25 000 Dutch patients with respect to histopathological quality of resection and postoperative morbidity and mortality.

## Patients and methods

### DSCA

Data were retrieved from the dutch surgical colorectal audit (DSCA), which was initiated by the Dutch Surgical Society to monitor and improve the quality of surgical care in colorectal cancer patients on a national level. The DSCA contains data on primary colorectal cancer resections registered by all 92 Dutch hospitals performing colorectal cancer surgery as from 2009 [11]. Recurrent colorectal cancer patients are not included in the database. The dataset shows a high level of completeness on most items and a case ascertainment of approximately 95 % when compared with the Netherlands Cancer Registry [12]. Details of the dataset regarding data collection and methodology have been published elsewhere [11]. Medical ethics committee approval was not required for this study as all patients and hospital information in the DSCA were anonymous.

### In-and exclusion criteria

All patients aged 18 years and older who underwent colonic and rectal cancer resection in the Netherlands between January 2010 and December 2012 were included in this study. Patients who underwent a transanal procedure were excluded from analysis.

### Outcomes

Primary outcome measures were number of harvested lymph nodes, circumferential rectal resection margin (CRM), CRM positivity, and completeness of resection in colon cancer. The CRM was considered positive if tumor cells were within 1 mm of the resection margin. Completeness of resection was defined as complete resection (resection with margins free of disease at histopathology, R0) or incomplete resection (margins positive for disease at histopathology, R1 or R2). R1 and R2 were taken together because of relatively small patient numbers with incomplete resection. Besides absolute number of harvested lymph nodes we used cut-offs of 10 and 12 lymph nodes. These are the cut-offs of the Dutch and US guidelines [13, 14]. Secondary outcome measures were postoperative complications and 30-day mortality.

Potential risk factors for adverse oncological or clinical outcome, including patient factors (age, sex, body mass index (BMI), ASA fitness grade, previous abdominal surgery), tumor factors (stage, location, preoperative tumor complications) and treatment factors (neoadjuvant therapy, type of surgical resection, operation technique, urgency of surgery, extent of resection), were extracted from the database. There is no information regarding adhesions in the database (e.g., incidence or severity of adhesions or adhesiolysis time).

### Statistical analysis

Univariable analyses were carried out to examine the association previous abdominal surgery and number of harvested lymph nodes, CRM, CRM positivity, completeness of resection, postoperative complications, and 30-day mortality. Multivariable logistic regression analyses were performed to correct for possible confounders. A manual stepwise model was used, with inclusion of variables with *P*  <  0·20. Clinically relevant variables, i.e., neoadjuvant therapy, were added to the statistical model. Conversion from laparoscopic to open technique and tumor localization were not included in the multivariate analysis because of the collinearity between prior operations and conversion, and between localization of the primary tumor and type of resection. Type of resection is highly influenced by the localization of the primary tumor, but the type of resection has a larger influence on clinical and histopathological outcome, especially in case of synchronous tumors.

Subgroup analysis regarding number of lymph nodes and percentage of resections with less than 10 and 12 lymph nodes was performed for colon and rectal cancer separately. Number of lymph nodes after colon and rectal cancer resection, percentage of colon and rectal cancer resections with less than 10 and 12 lymph nodes, incomplete colon cancer resection and CRM and CRM positivity were analyzed in subgroups of different types of abdominal surgery in history as defined in the database: colorectal surgery (including appendectomy), urogenital surgery, hepatobiliary surgery (including cholecystectomy), upper gastrointestinal surgery (including pancreatic surgery), or other abdominal surgery not otherwise specified. These subgroups of previous surgery were analyzed because reported incidences of adhesion-related and adhesiolysis-related complications differ between different anatomical locations of prior surgery [2, 15]. This subgroup analysis was performed in the cohort 2011–2012, because of a high level of missing data on location of prior surgery in patients operated in 2010. Subgroup analysis by magnitude of the previous operation was not possible because this item is not registered in the database.

Results are reported as odds ratios with 95 % confidence intervals. All data were analyzed using SPSS for Windows version 20.0 software (SPSS, Chicago, IL). We excluded per analysis those cases with missing data.

## Results

27,341 Colorectal cancer patients were included in the database from 2010 to 2012. After excluding patients younger than 18 years (*n* = 17), transanal procedures (*n* = 344), and patients with missing data of prior abdominal surgery (*n* = 259), 26,721 patients were eligible for inclusion. 9042 Patients (33.8 %) had undergone one or more previous abdominal operations,17,679 patients (66.2 %) had no prior abdominal surgery.

### Baseline characteristics

Baseline characteristics for the groups with and without prior abdominal surgery are presented in Table 1. All data significantly differed between groups due to large patient numbers. Clinically relevant differences were found for age and sex. Mean age was 71.3 in the prior abdominal surgery group compared to 68.4 in the no prior abdominal surgery group. The prior abdominal surgery group comprised 41.9 % male patients compared to 61.3 % in the no prior abdominal surgery group.

**Table 1:** Baseline characteristics Cohort 2010–2012

Prior abdominal operations	Yes (*N* = 9042)	No (*N* = 17,679)	*P* Value
Age	71.3 (±10.8)	68.4 (±11.3)	<0.001
Sex (%)	41.9 % male	61.3 % male	<0.001
ASA (%)			
I	15.5	23.0	<0.001
II	58.2	55.1	
III	24.8	20.0	
IV	1.5	1.8	
V	0.1	0.1	
Urgency operation (%)			
Elective	85.9	83.8	<0.001
Elective after stent placement	0.7	0.6	
Urgent	7.4	8.0	
Emergency	5.9	7.5	
Unknown	0.2	0.1	
Tumor localization (%)			
Caecum	15.9	13.3	<0.001
Appendix	0.8	0.4	
Ascending colon	14.6	12.4	
Hepatic flexure	4.8	4.4	
Transverse colon	6.6	5.0	
Splenic flexure	2.3	2.4	
Descending colon	4.7	4.3	
Sigmoid colon	25.3	28.8	
Rectum	24.9	29.0	
Neoadjuvant Therapy (%) (rectal cancer, *N* = 6457)	N = 1921	*N* = 4536	0.26
No	3.4	2.7	
Short course	55.2	54.1	
Long course	5.9	5.8	
Chemoradiation	35.5	37.4	
Operation technique (%)			
Open	60.6	54.2	<0.001
Laparoscopic	39. 4	45.8	
Conversion in laparoscopy (*n* = 8589) (%)			
No	76.5	82.6	<0.001
Early (<30 min)	12.5	8.5	
Late (>30 min)	11.1	9.0	
Reason for conversion %			
(*N* = 1406)			<0.001
Advanced tumor	14.3	26.9	
Accessibility	79.1	65.4	
Peroperative complication	6.7	7.7	
Type of resection %			
Ileocaecal	1.1	0.9	<0.001
Resection/appendicectomy	36.1	30.7	
Right hemicolectomy	2.7	1.9	
Transverse colectomy	7.8	7.7	
Left hemicolectomy	40.6	47.3	
(Low)Anterior	1.7	1.4	
Resection/sigmoidectomy	7.6	8.5	
Subtotal colectomy	0.8	0.6	
Abdominoperineal Resection	1.4	1.0	
Proctocolectomy			
Other			
T stage (pathol) (%)			
1	7.1	6.1	0.001
2	20.8	19.6	
3	55.7	57.6	
4	13.0	13.5	
X	2.1	1.8	
0	1.3	1.4	
N stage (pathol) %			
0	60.3	57.5	<0.001
1	24.3	25.4	
2	13.8	16.1	
X	1.6	1.0	

### Histopathological and clinical outcome

Mean number of lymph nodes in the histopathology specimen was 15.2 in the group with and 15.6 in the group without prior abdominal surgery (Table 2). Number of lymph nodes was less than 10 and 12 in 20.7 and 35.8 % of patients with prior abdominal surgery compared to 17.8 and 32.8 % in patients without prior abdominal surgery. For colonic resection the percentage of patients with less than 10 and 12 lymph nodes was higher in the prior surgery compared to the no prior surgery group. For rectal resection differences were not significant after adjustment for other variables (Table 2). No differences were found in completeness of colonic resection, mean circumferential rectal resection margin and CRM positivity.

**Table 2:** Histopathological and clinical outcome, cohort 2010–2012

	Prior abdominal operations *N* = 8949^b^	No prior abdominal operations *N* = 17,534^b^	Crude OR/mean difference (95 % CI)	Adjusted OR/mean difference (95 % CI)^a^
Number of lymph nodes	15.2	15.6	0.39 (0.11–0.66)*	0.37 (0.14–0.60)*
Colon cancer	16.1	16.6	0.55 (0.200.90)*	0.43 (0.15–0.71)*
Rectal cancer	12.5	13.0	0.46 (0.11–0.81)*	0.14 (−0.23 to 0.50)
<10 Lymph nodes (% pts)	20.7	17.8	1.20 (1.13–1.28)*	1.17 (1.09–1.26)*
Colon cancer	16.5	13.4	1.28 (1.18–1.39)*	1.22 (1.11–1.33)*
Rectal cancer	32.9	28.7	1.22 (1.10–1.36)*	1.09 (0.96–1.23)
<12 Lymph nodes (% pts)	35.8	32.8	1.14 (1.08–1.20)*	1.10 (1.04–1.17)*
Colon cancer	30.7	27.6	1.16 (1.09–1.24)*	1.10 (1.02–1.18)*
Rectal cancer	50.8	45.6	1.23 (1.11–1.36)*	1.11 (0.99–1.24)
Incomplete resection colon cancer (% pts)	3.3	3.8	0.87 (0.73–1.02)	0.92 (0.77–1.11)
Circumferential rectal resection margin (mm)	11.0	11.1	0.111 (−0.56 to 0.77)	0.10 (−0.82 to 1.02)
CRM positivity (% pts)	10.2	9.1	1.14 (0.94–1.38)	1.12 (0.90–1.40)
Complications (% pts)	34.5	32.1	1.11 (1.05–1.17)*	1.14 (1.07–1.21)*
Colon cancer	33.2	29.7	1.17 (1.10–1.25)*	1.18 (1.10–1.26)*
Rectal cancer	38.0	37.9	1.01 (0.91–1.11)	1.04 (0.92–1.17)
30-day Mortality (% pts)	4.7	3.8	1.24 (1.09–1.40)*	1.01 (0.88–1.17)
Colon cancer	5.3	4.3	1.24 (1.08–1.42)*	1.01 (0.86–1.18)
Rectal cancer	2.7	2.6	1.04 (0.76–1.41)	1.02 (0.70–1.47)

* *P* < 0.05

^a^Adjusted for male sex, age, ASA fitness grade, type of surgical resection, T stage at histopathology, N stage at histopathology, urgency of surgery, operation technique and neoadjuvant chemotherapy

^b^Missing data excluded per analysis

There was a small but significant increase in percentage of patients with postoperative complications after prior surgery (34.5 and 32.1 %; adjusted OR 1.14 (95 % CI 1.07–1.21). For colonic resection the percentage of patients with complications was higher in the prior surgery compared to the no prior surgery group. For rectal resection no differences was found (Table 2). 30-day mortality was 0.9 percent higher in patients with (4.7 %) compared to those without prior abdominal surgery (3.8 %). This difference was not significant after adjustment for other variables.

### Histopathological outcome by type of prior operation

Histopathological results divided by type of prior abdominal surgery, i.e., colorectal surgery, urogenital surgery, hepatobiliary surgery, upper gastrointestinal surgery and other abdominal operations are shown in Table 3 for colonic resection and in Table 4 for rectal resection. Prior upper gastrointestinal and urogenital operations did not compromise the quality of oncological resection as reflected by number of lymph nodes, resection margins and completeness of resection, for both colonic and rectal resections. Prior colorectal resection significantly decreased the number of lymph nodes in colon specimens. There was a trend towards a higher percentage of patients with less than 12 lymph nodes in the specimen (28.9 vs 25.5 %, *P* = 0.058). An almost two-third increase in patients with CRM positivity of rectal specimens was found (12.5 % prior colorectal resection and 7.6 no prior colorectal resection). Prior hepatobiliary surgery and prior other abdominal surgery were associated with increased percentages of patients with less than 10 lymph nodes by 24 and 26, respectively, for colonic resection. For rectal resection, prior hepatobiliary surgery increased the percentage of patients with less than 12 lymph nodes by 21. No significant effects of other abdominal surgery were found for rectal resections (Table 4).

**Table 3:** Histopathological outcome after colon cancer surgery according to type of prior abdominal surgery, cohort 2011–2012

	Prior colorectal operations *N* = 1832^b^	No prior abdominal operations *N* = 10,404^b^	Crude OR/mean difference (95 % CI)	Adjusted OR/mean difference (95 % CI)^a^
Number of lymph nodes	16.3	17.1	0.80 (0.33–1.27)*	0.61 (0.14–1.08)*
<10 lymph nodes (% pts)	14.2	11.9	1.23 (1.06–1.42)*	1.11 (0.94–1.30)
<12 lymph nodes (% pts)	28.9	25.5	1.19 (1.06–1.33)*	1.12 (1.00–1.27)**
Incomplete resection (% pts)	6.2	3.8	1.66 (1.14–2.42)*	1.30 (0.90–1.89)

	Prior urogenital operations *N* = 1790^b^	No prior abdominal operations *N* = 10,440^b^	Crude OR/mean difference (95 % CI)	Adjusted OR/mean difference (95 % CI)^a^
Number of lymph nodes	16.8	17.0	0.23 (−0.24 to 0.71)	0.25 (−0.25 to 0.74)
<10 Lymph nodes (% pts)	13.0	12.1	1.08 (0.93–1.26)	1.09 (0.92–1.29)
<12 Lymph nodes (% pts)	25.9	26.1	0.99 (0.88–1.11)	0.97 (0.86–1.10)
Incomplete resection (% pts)	3.3	3.6	0.91 (0.68–1.20)	1.03 (0.76–1.39)

	Prior hepatobiliary operations *N* = 922^b^	No prior abdominal operations *N* = 11,317^b^	Crude OR/mean difference (95 % CI)	Adjusted OR/mean difference (95 % CI)^a^
Number of lymph nodes	16.2	17.0	0.77 (0.13–1.40)*	0.40 (-0.24 to 1.03)
<10 Lymph nodes (% pts)	14.9	12.0	1.28 (1.06–1.54)*	1.28 (1.04–1.58)*
<12 Lymph nodes (% pts)	28.2	25.9	1.12 (0.97–1.30)	1.09 (0.92–1.28)
Incomplete resection (% pts)	3.7	3.5	1.04 (0.73–1.49)	1.15 (0.78–1.71)

	Prior upper gastrointestinal operations *N* = 200^b^	No prior abdominal operations *N* = 10,928^b^	Crude OR/mean difference (95 % CI)	Adjusted OR/mean difference (95 % CI)^a^
Number of lymph nodes	21.7	17.0	−4.68 (−6.58 to −2.78)	−0.25 (−1.60 to 1.11)
<10 Lymph nodes (% pts)	16.0	12.0	1.40 (0.95–2.05)	1.13 (0.74–1.73)
<12 Lymph nodes (% pts)	28.0	25.9	1.11 (0.82–1.52)	0.91 (0.65–1.27)
Incomplete resection (% pts)	3.3	3.6	0.94 (0.44–2.00)	1.08 (0.45–2.58)

	Prior other abdominal operations *N* = 815^b^	No prior abdominal operations *N* = 11,296^b^	Crude OR/mean difference (95 % CI)	Adjusted OR/mean difference (95 % CI)^a^
Number of lymph nodes	16.7	17.0	0.28 (−0.39 to 0.95)	0.12 (−0.55 to 0.79)
<10 Lymph nodes (% pts)	15.1	12.0	1.30 (1.06–1.59)*	1.28 (1.02–1.59)*
<12 Lymph nodes (% pts)	29.2	25.8	1.18 (1.01–1.38)*	1.16 (0.98–1.37)
Incomplete resection (% pts)	3.7	3.6	1.04 (0.71–1.53)	1.06 (0.71–1.60)

^a^Adjusted for male sex, age, ASA fitness grade, type of surgical resection, T stage at histopathology, N stage at histopathology, urgency of surgery, operation technique and neoadjuvant chemotherapy

^b^Missing data excluded per analysis

* *P* < 0.05

** *P* = 0.058

**Table 4:** Histopathological outcome after rectal cancer surgery according to type of prior abdominal surgery, cohort 2011–2012

	Prior colorectal operations *N* = 588^b^	No prior abdominal operations *N* = 3654^b^	Crude OR/mean difference (95 % CI)	Adjusted OR/mean difference (95 % CI)^a^
Number of lymph nodes	12.8	13.3	0.42 (−0.19 to 1.03)	0.22 (−0.41 to 0.85)
<10 lymph nodes (% pts)	30.4	26.2	1.23 (1.02–1.49)*	1.11 (0.89–1.38)
<12 lymph nodes (% pts)	47.4	43.9	1.15 (0.97–1.37)	1.03 (0.84–1.25)
Circumferential margin (mm)	10.0	10.8	0.83 (−0.12 to 1.77)	0.64 (−0.37 to 1.65)
CRM positivity (% pts)	12.5	7.6	1.74 (1.29–2.33)*	1.70 (1.21–2.39)*

	Prior urogenital operations *N* = 531^b^	No prior abdominal operations *N* = 3711^b^	Crude OR/mean difference (95 % CI)	Adjusted OR/mean difference (95 % CI)^a^
Number of lymph nodes	12.7	13.3	0.56 (−0.08 to 1.21)	0.22 (−0.49 to 0.93)
<10 lymph nodes (% pts)	29.2	26.5	1.14 (0.94–1.40)	1.00 (0.78–1.28)
<12 lymph nodes (% pts)	48.2	43.8	1.19 (1.00–1.43)	1.13 (0.91–1.41)
Circumferential margin (mm)	12.1	10.5	−1.59 (−2.60 to −0.59) *	−1.80 (−2.95 to −0.65) *
CRM positivity (% pts)	5.2	8.7	0.58 (0.37–0.90)	0.71 (0.44–1.16)

	Prior hepatobiliary operations *N* = 271^b^	No prior abdominal operations *N* = 3965^b^	Crude OR/mean difference (95 % CI)	Adjusted OR/mean difference (95 % CI)^a^
Number of lymph nodes	12.5	13.3	0.76 (−0.11 to 1.63)	0.59 (−0.29 to 1.47)
<10 Lymph nodes (% pts)	32.1	26.5	1.31 (1.01–1.71)*	1.21 (0.90–1.63)
<12 Lymph nodes (% pts)	53.1	43.8	1.45 (1.14–1.86)*	1.32 (1.01–1.74)*
Circumferential margin (mm)	9.9	10.8	0.83 (−0.47 to 2.14)	0.60 (−0.78 to 1.98)
CRM positivity (% pts)	6.1	8.4	0.71 (0.42–1.21)	0.53 (0.28–1.01)

	Prior upper gastrointestinal operations *N* = 81^b^	No prior abdominal operations *N* = 3785^b^	Crude OR/mean difference (95 % CI)	Adjusted OR/mean difference (95 % CI)^a^
Number of lymph nodes	14.2	13.3	−0.91 (−2.46 to 0.65)	−1.13 (−2.70 to 0.44)
<10 Lymph nodes (% pts)	21.0	26.2	0.75 (0.44–1.28)	0.63 (0.34–1.18)
<12 Lymph nodes (%)	35.8	43.8	0.72 (0.45–1.13)	0.67 (0.40–1.12)
Circumferential margin (mm)	12.2	10.9	−1.36 (−3.88 to 1.15)	−1.55 (−4.21 to 1.11)
CRM positivity (% pts)	10.6	7.7	1.43 (0.64–3.15)	1.62 (0.70–3.77)

	Prior other abdominal operations *N* = 316^b^	No priorabdominal operations *N* = 3885^b^	Crude OR/mean difference (95 % CI)	Adjusted OR/mean difference (95 % CI)^a^
Number of lymph nodes	13.0	13.2	0.25 (−0.56 to 1.06)	0.28 (−0.57 to 1.13)
<10 Lymph nodes (% pts)	27.5	26.7	1.04 (0.81–1.35)	1.09 (0.81–1.48)
<12 Lymph nodes (% pts)	44.9	44.2	1.03 (0.82–1.30)	0.98 (0.75–1.28)
Circumferential margin (mm)	11.3	10.7	−0.68 (−1.93 to 0.57)	−0.68 (−2.06 to 0.69)
CRM positivity (%)	9.4	8.2	1.73 (1.29–2.31)*	1.44 (0.89–2.36)

^a^Adjusted for male sex, age, ASA fitness grade, type of surgical resection, T stage at histopathology, N stage at histopathology, urgency of surgery, operation technique and neoadjuvant chemotherapy

^b^Missing data excluded per analysis

* *P* < 0.05

## Discussion

Prior abdominal surgery jeopardizes subsequent abdominal surgical procedures. In the large Dutch Surgical Colorectal Cancer Audit (DSCA) prospective database of colorectal cancer patients increased postoperative complications were demonstrated after prior abdominal surgery. More importantly, prior abdominal surgery had negative effects on histopathological outcome parameters. A higher risk of inadequate numbers of harvested lymph nodes was demonstrated for colonic resections. Prior colorectal surgery was associated with an almost two-third increase of positive circumferential resection margins in rectal cancer patients.

Results of the present study are in agreement with an earlier report of a higher morbidity rate after repeat surgery [6]. In a case-matched study of laparoscopic intestinal resection even a doubling of the incidence of postoperative complications was found after previous midline laparotomy [1]. The DSCA database does not contain information on the extent of prior operations, but only a gross differentiation of anatomical locations where patients had their previous abdominal surgery. It is likely that the prior abdominal surgery group partly consists of laparoscopic or minor surgical procedures such as appendectomy and cholecystectomy, which may account for the relatively small effect on postoperative morbidity found in the present study.

Two small retrospective series demonstrated no impact of prior abdominal surgery on histopathological outcome parameters in colorectal cancer. The number of lymph nodes in colon specimens resected via minilaparotomy was similar between 76 patients with prior abdominal surgery and 187 patients without prior surgery [10]. Comparison of lymph node numbers and resection margins after laparoscopic colorectal resection in 16 patients with and 44 patients without previous abdominal surgery also revealed no differences [9]. These different results can be explained by the small number of patients included in both studies. The low mean number of lymph nodes, in both studies less than 10, raises doubts about the quality of surgery or histopathological examination.

Multiple studies have been published evaluating risk factors for circumferential margin positivity in rectal cancer surgery. Well recognized risk factors for increased CRM positivity are higher T and N stage, male sex and absence of preoperative chemoradiation [16–19]. Prior abdominal surgery has never been taken into account in previous studies. Since the mesorectal fascia is a retroperitoneal plane, CRM will not be directly affected by intraperitoneal adhesions. However, when the lower abdomen has been explored before surgeons can experience difficulty gaining access to the pelvis. The association of prior colorectal surgery with increase in positive CRM is therefore most likely explained by more challenging surgery and compromised access to the pelvic area due to intraperitoneal adhesions.

The major strength of this study is the use of a very large prospective, complete and validated dataset. However, there are limitations, because the database does not contain descriptions of the presence or severity of adhesions, nor whether adhesiolysis was performed during surgery. Adhesiolysis has demonstrated to increase morbidity and mortality in previous studies [6, 7] and adhesions as a factor for a lower quality of colorectal resection specimens is suggested given the significant findings for prior surgery. The effect of postoperative adhesions on histopathological outcome might even have been underestimated, because a small portion of patients with prior abdominal surgery do not have adhesions [2–5]. On the other hand, intra-abdominal adhesions also occur in patients without prior abdominal surgery. However, the incidence of these adhesions is less than 30 % [5] and they are mostly low-grade, easy to separate and do not require a lengthy adhesiolysis [6].

Some known risk factors for adverse histopathological outcome, such as distance to the nearest bowel resection plane and failure to use a pathology template were not available in the DSCA database [8]. The pathology template for colorectal cancer was introduced in the Netherlands in 2009 and is generally used.

Prior abdominal surgery was not specified in the database except for the ‘anatomical location’. Particularly, magnitude (i.e., laparoscopic or minimal invasive approach) of the previous operations and intra-abdominal complications, e.g., postoperative peritonitis could not be assessed. Differences in results between open and minimal invasive prior surgery is expected as there is increasing evidence for a lower risk of adhesion-related complications after laparoscopic surgery [20]. Postoperative peritonitis may render a minimal invasive operation into a very adhesiogenic surgical procedure. In our own series of consecutive elective colorectal operations 15 % of patients who needed adhesiolysis had suffered from previous intra-abdominal infection [6]. The lack of specific information on prior surgery does not make the negative effect of prior abdominal surgery on outcome of colorectal cancer surgery less plausible. At most, we can assume that the negative impact is greater when prior abdominal operations are major. Eight-two percent of patients with prior urogenital operations were women and diagnostic laparoscopy and laparoscopic tubal ligation were probably the most common procedures. Female gender and a minimally adhesiogenic procedure in history may explain the association of prior urogenital procedures with a larger circumferential margin in rectal resection.

Long-term oncological outcome, overall, and disease-free survival are not available in the DSCA database. However, previous studies have shown that an inadequate number of lymph nodes evaluated is associated with an impaired outcome [21, 22], and CRM positivity increases local recurrence risk [23]. Additionally, the higher incidence of postoperative complications might worsen long-term oncological outcome [24].

With higher life-expectancy and advances in surgical technique the incidence of repeat abdominal surgery has increased. Since adhesion formation is a possible reason for our findings, routine use of anti-adhesive barriers particularly in initial colorectal and hepatobiliary surgery could potentially benefit outcome of reoperations in the same anatomical areas. In a recent systematic review, we showed that hyaluronate carboxymethylcellulose has the potential to alleviate the incidence of adhesion-related complications in colorectal surgery [25]. Also in two-stage liver surgery hyaluronate carboxymethylcellulose was shown to reduce operation time [26]. The potentially beneficial effect of anti-adhesives on histopathological results of oncological resections should be taken into account in future studies on adhesion prevention.

This present study addresses, the negative effect of prior abdominal surgery on the outcome of colorectal cancer surgery. Surgeons should be aware of this effect when performing an oncological resection in patients with abdominal surgery in history to dissect the right planes and obtain sufficient amounts of lymph nodes not compromising the extent of resection. The completeness and quality of preoperative patient informed consent may benefit from the results of this study.
